# “Double rings” En-bloc technique with early anatomical apical release in thuflep for the patients with large glands (≥ 80 mL): the outcomes from single center

**DOI:** 10.1186/s12894-025-01851-2

**Published:** 2025-07-05

**Authors:** Kai Zhang, Kunlin Yang, Chao Zuo, Yang He, Zhicun Li, Xinyan Che, Yisen Meng

**Affiliations:** 1 Department of Urology, Peking University First Hospital, No. 8 Xishiku St, Xicheng District, Beijing, 100034 China; 2 Institute of Urology, Peking University, Beijing, No. 8 Xishiku St, Xicheng District, 100034, China; 3 National Urological Cancer Center, Beijing, No. 8 Xishiku St, Xicheng District, 100034 China; 4 Department of Urology, Beijing Miyun District Hospital, Beijing, China

**Keywords:** Thulium, Laser enucleation, Large prostate, Apical release, En-bloc

## Abstract

**Background and purpose:**

We aimed to describe the technique and outcomes of “Double Rings” En-bloc thulium fiber laser enucleation of the prostate (ThuFLEP) with early anatomical apical release in benign prostatic hyperplasia (BPH) with large glands (≥ 80 mL).

**Patients and methods:**

A total of 68 BPH patients with large glands (≥ 80 mL) who received our technique performed by a single surgeon were enrolled between January 2021 and January 2022. The parameter set of thulium fiber laser was 1.5 J/40 Hz (60 W). Enucleation was performed using the “Double Rings” technique. The first ring was created by circumferentially incising the urethral mucosa around the adenoma at the apex. The second ring was formed by dissecting the bladder neck at 12 o’clock position, which served as a landmark for guiding further dissection. The adenoma was then pushed into the bladder, and the bleeding points of the prostatic fossa were coagulated with a thulium fiber laser to ensure hemostasis. We analyzed patient demographic, perioperative and two-year follow-up data.

**Results:**

Mean preoperative prostate volume was 135 ml (range, 80–275). Mean preoperative International Prostate Symptom Score (IPSS) was 21.1 (range, 17.8–29.6). Mean preoperative quality of life (QoL) score was 4.7 (range, 3–5). Mean operative time was 88.6 min (range, 40–199). Mean enucleated tissue weight was 70.8 g (range, 30–162). The rate of postoperative complication was 11.8% (2 fevers and 6 urinary tract infections), which were manageable. No blood transfusion. IPSS, QoL score, peak urinary flow rate, and postvoid residual urine volume were all significantly improved (*p* < 0.01). Six patients had transient early stress urinary incontinence (SUI), but all recovered in 3 months. Over a follow-up of 38.2 months (range: 28–45 months), no urethral stricture or bladder neck contracture were detected. No patient developed permanent SUI.

**Conclusions:**

“Double Rings” En-bloc technique with early anatomical apical release in ThuFLEP seems to be a safe and effective approach for BPH patients with large glands. However, multi-center and large sample studies are still needed.

**Supplementary Information:**

The online version contains supplementary material available at 10.1186/s12894-025-01851-2.

## Introduction

Although various minimally invasive treatments have been developed for benign prostatic hyperplasia (BPH), surgical options remain limited for patients with large-volume prostates [1]. Transurethral resection of the prostate (TURP), a classical approach, is associated with a higher risk of complications in these cases, while open simple prostatectomy, although effective, carries significant morbidity. To overcome these limitations, laser-assisted anatomical endoscopic enucleation of the prostate (L-EEP) has been introduced and widely adopted, offering excellent outcomes with an acceptable safety profile [2]. Meanwhile, advances in surgical technology have also led to increased adoption of waterjet ablation therapy or minimal invasive simple prostatectomy for large prostates [1, 3].

Most L-EEP studies have focused on holmium laser enucleation of the prostate (HoLEP), while reports on thulium fiber laser enucleation of the prostate (ThuFLEP) remain relatively scarce [4]. Large prostates are often characterized by increased vascularity. Although the thulium fiber laser (TFL) lacks the mechanical shockwave effect of the holmium laser for developing the surgical plane, it offers precise cutting, effective coagulation, and excellent hemostasis with minimal tissue penetration [4]. We have found that our “Double Grooves–Double Rings” technique can facilitate rapid mastery of ThuFLEP, especially for novice surgeons [5]. Therefore, in this study, we evaluated the efficacy and safety of the “Double Rings” En-bloc technique with early anatomical apical release in ThuFLEP for patients with large prostates (≥ 80 mL).

## Materials and methods

### Patients and methods

A prospectively maintained database of all ThuFLEP cases performed in our department by a single surgeon between January 2021 and January 2022 was reviewed. We finally selected 68 patients with large gland (≥ 80 mL) who underwent “Double Rings” En-bloc technique with early anatomical apical release for BPH. The data were then retrospectively extracted and analysed. All patients were preoperatively evaluated with medical history, International Prostate Symptom Score (IPSS), ultrasonography, serum prostate-specific antigen, peak urinary flow rate (Qmax), and postvoid residual urine volume (PVR) before surgery and had indications for surgery. The patients with clinical suspicion of prostate cancer underwent preoperative prostate biopsy.

### Equipment and preoperative preparation

Laser energy was delivered by a TFL device (Raykeen, China) through a 550-mm reusable laser fiber (Raykeen Laser products). The parameter set was 1.5 J/40 Hz, and a routine power output of 60 W was used. Enucleation was performed using a 26-French continuous-flow laser resectoscope combined with morcellator (Hawk, China). Normal saline was used as the irrigation fluid. Procedure was performed under general anesthesia with the patient in lithotomy position.

### Description of surgical technique

**Fig. 1:**
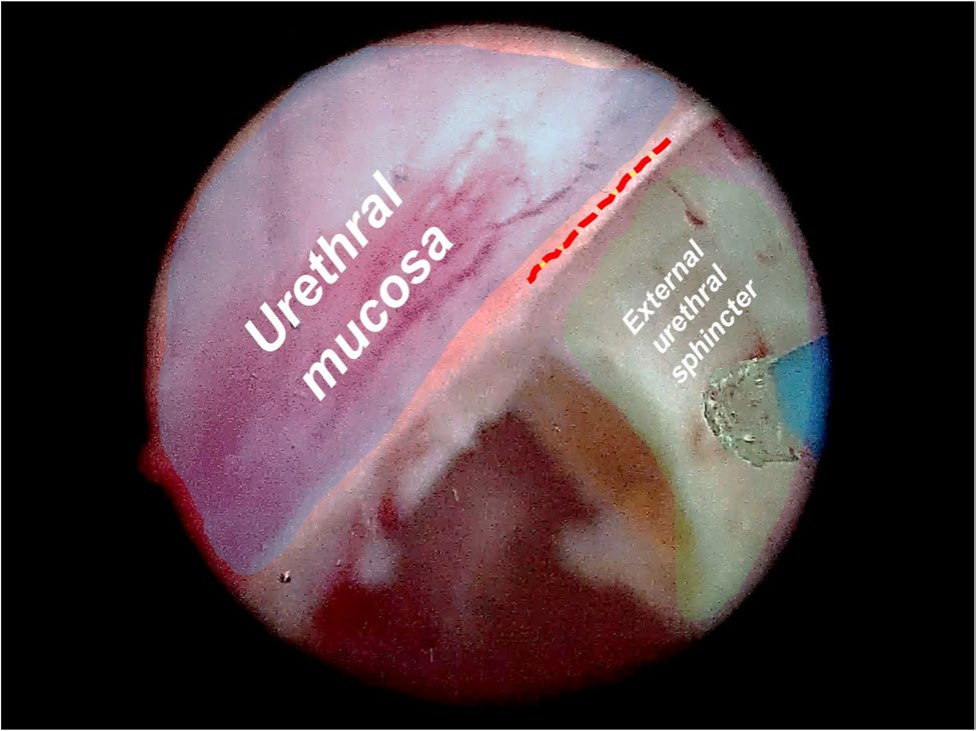
The anatomical relationship between the urethral mucosa and the external urethral sphincter. The red dotted line indicates that the urethral mucosa be cut by laser along the edge of the urethral sphincter

**Fig. 2:**
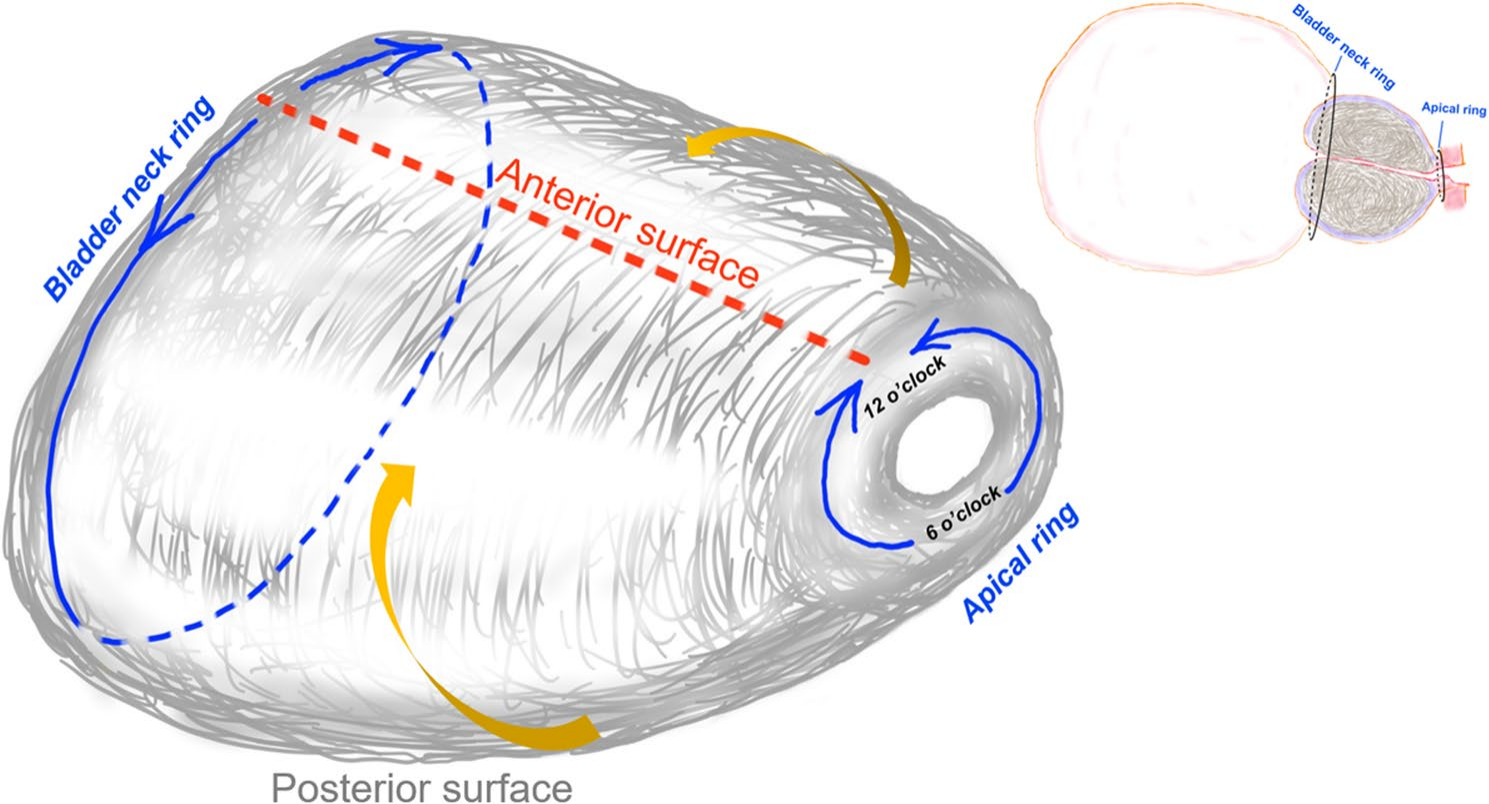
“Double Rings” En-bloc technique in thulium fiber laser enucleation of the prostate. The term of “apical ring” refers to the early anatomical apical release which is the first step. The circumferential incisions are made between the urethral sphincter and the prostatic adenoma, spanning from 6 o’clock to 12 o’clock positions. The yellow arrows indicate dissection of bilateral surfaces from posterior to anterior surface. Bladder neck ring means that the bladder neck is reached at the 12 o’clock position and dissected away from the adenoma using an up-down technique. An orange dotted line represents a potential groove that can be created at this position as a landmark for accessing the bladder neck when dealing with elongated adenomas or thick anterior tissue

Enucleation was initiated at the prostatic apex. An inverted U-shaped incision was made just proximally to the verumontanum, and the surgical capsule was identified through gentle blunt dissection. After visualizing the urethral sphincter, circumferential incisions between the sphincter and the left prostatic adenoma were performed from the 6 o’clock position in an anticlockwise direction. Simultaneously, the urethral mucosa was incised using the laser along the edge of the sphincter (Fig. 1). The same steps were then repeated on the right apex, completing what we defined as the first ring (Fig. 2, Supplementary Video 1).

Once apical dissection was anatomically completed, both lateral lobes were enucleated from the posterior plane toward the anterior surface (Fig. 2). The bladder neck was then reached at the 12 o’clock position and dissected away from the adenoma using what we refer to as the “up-down” technique, completing the second ring (Fig. 2). In cases where the adenoma was excessively long or the anterior tissue was particularly thick, a groove was created at the 12 o’clock position to facilitate identification of the bladder neck as a reliable anatomical landmark (Fig. 2).

Finally, the whole adenoma was pushed into the bladder through the 6 o’clock position of the bladder neck. Before morcellation, the bleeding points of prostatic fossa were coagulated using TFL to obtain a clear endoscopic view. Finally, a quick check was performed with the necessary coagulation using TFL.

### Postoperative management and follow-up

The Foley catheter was routinely removed 2–6 days after surgery. Blood loss was estimated by hemoglobin (Hb) drop on postoperative day 1. The enucleated tissue was analyzed histopathologically. The significant storage symptoms were treated with medical therapy (e.g. solifenacin succinate or mirabegron). Patients were discharged home after successful voiding. Perioperative data (i.e. operative time, weight of enucleated prostate tissue, Hb drop, catheterization time, postoperative hospitalization, complications) were recorded. Complications were evaluated by Clavien-Dindo (CD) classification system. All operated patients on follow-up were evaluated for IPSS, QoL, Qmax, and PVR at 3 months, 6 months, 1 year and 2 years after surgery. Mean IPSS, QoL, Qmax, and PVR were compared preoperatively and postoperatively by paired t-test with *p* < 0.05 considered statistically significant.

## Results

The median age of patients was 69 years (range, 56–88). Approximately 76% of patients had urinary retention. Patients’ baseline characteristics are shown in Table 1. Mean preoperative prostate volume was 135 ml (range, 80–275). Mean operative time was 88.6 min (range, 40–199). The mean weight of enucleated tissue was 70.8 g (range, 30–162). Mean postoperative Hb drop was 1.7 g/dL (range, 0-4.3) on postoperative day 1. Mean catheterization time was 2.5 days (range, 2–6). Mean postoperative hospitalization time was 4 days (range, 3–7). Two patients had incidental prostate cancer confirmed by postoperative histopathology and received active surveillance. Eight patients had postoperative complications including 2 fevers (CD grade I) and 6 urinary tract infections (CD grade II). No patient received blood transfusion.

**Table 1:** Patients’ characteristics, preoperative and perioperative data

	**Results**
Total number of the patients, n	68
The median age, years (range)	69 (56-88)
The mean BMI, kg/m^2^(range)	24.1 (17.8-29.6)
Indications of surgery, n (%)	
LUTS	14 (20.6%)
Urinary retention	52 (76.5%)
Hematuria	2 (2.9%)
Preoperative prostate volume (ml), mean (range)	135.0 (80-275)
Preoperative PSA (ng/mL), mean (range)	10.08 (1.03-64.83)
Preoperative IPSS, mean (range)	21.1 (8-35)
Preoperative QoL score, mean (range)	4.7 (3-5)
Preoperative Qmax (ml/sec), mean (range)	7.6 (2.3-18.6)
Preoperative PVR (mL), mean (range)	75.1 (0-397)
Preoperative Hb (g/dL), mean (range)	14.1 (10.7-16.1)
ASA classification	
1	1 (1.5%)
2	47 (69.1%)
3	19 (27.9%)
4	1 (1.5%)
Operative time (min), mean (range)	88.6 (40-199)
Enucleated prostatic tissue weight (g), mean (range)	70.8 (30-162)
Postoperative Hb POD 1 (g/dL), mean (range)	12.5 (9.0-15.8)
Hb drop POD 1 (g/dL), mean (range)	1.7 (0-4.3)
Catheterization time (day), mean (range)	2.5 (2-6)
Postoperative hospitalization (day), mean (range)	4.0 (3-7)
Presence of incidental prostate cancer	2 (2.9%)
Postoperative complications, CD grade, n (%)	
Fever (grade I)	2 (2.9%)
UTI (grade II)	6 (8.8%)

*BMI* body mass index, *LUTS* lower urinary tract symptoms, *PSA* prostate-specific antigen, *IPSS* International Prostate Symptom Score, *QoL* quality of life, *Qmax* peak urinary flow rate, *PVR* postvoid residual urine volume, *Hb* hemoglobin, *ASA* American Society of Anesthesiologists, *POD* postoperative day, *CD* Clavien-Dindo, *UTI* urinary tract infection

IPSS, QoL score, Qmax, and PVR all improved significantly (*p* < 0.01) after surgery and remained stable during follow-up (Table 2). One patient had temporary urinary retention immediately after catheter removal but recovered. Six patients had mild and transient early stress urinary incontinence (SUI). However, they reported that the incontinence did not significantly affect their quality of life, and all patients achieved full recovery within three months. Over a follow-up of 38.2 months (range: 28–45 months), no urethral stricture or bladder neck contracture were detected. No patient developed permanent SUI up to the final follow up.

**Table 2:** Preoperative data and follow-up changes after surgery

	Mean IPSS	Mean QoL score	Mean Qmax (ml/sec)	Mean PVR (mL)
Preoperative (68)	21.1 (8–35)	4.7 (3–5)	7.6 (2.3–18.6)	75.1 (0-397)
3 months (66)	6.4 (0–19)	1.4 (1–5)	23.3 (6.3–52.3)	25.0 (0-104)
6 months (63)	5.3 (0–17)	0.8 (0–4)	25.2 (5.9–54)	22.0 (0–89)
1 year (62)	5.3 (0–16)	0.6 (0–3)	25.4 (10.3–42.3)	21.8 (0–90)
2 years (51)	5.0 (0–18)	0.6 (0–4)	29.0 (9.2–48.5)	21.2 (0–82)

*PSA* prostate-specific antigen, *IPSS* International Prostate Symptom Score, *QoL* quality of life, *Qmax* peak urinary flow rate, *PVR* postvoid residual urine volume

## Discussion

Transurethral laser enucleation of the prostate has been shown to be a safe and effective procedure for symptomatic BPH regardless of prostate size [1, 2]. However, transurethral enucleation of a large prostate still has inherent challenges, including the prolonged operative time, increased risk of complications, and increased demands on surgeons. For the patients with a large prostate, both operative time and blood loss are important factors to consider, as the longer the operative time and the more blood loss, the greater the risk of adverse events [6].

Theoretically, TFL has the advantage of better absorption in water than holmium laser, which provides a good coagulation function and precise cutting effect [7]. In addition, TFL is the best choice due to its versatility, including vaporization, resection, enucleation, and vapoenucleation [7]. ThuFLEP may shorten the learning curve, achieving proficiency after performing 8–16 cases, whereas HoLEP typically requires 50–60 procedures [4]. Our study also showed that a novice can preliminarily master ThuFLEP after performing 14 cases using “Double Grooves‑Double Rings” two/three-lobe technique [5].

High vascular density is common in large prostates [1, 6], so the good coagulation function of the laser can reduce the blood loss, achieve effective bleeding control, and provide a clear endoscopic view for subsequent safe morcellation. We routinely use TFL to re-coagulate the prostatic fossa after morcellation. Some surgeons may prefer to use electro-coagulation [8]. In our experience, TFL is sufficient to perform coagulation without changing the additional instruments.

Furthermore, large prostates are more likely to have multiple hyperplastic nodules, which can affect the surgeon’s judgement in finding the correct surgical capsule or plane [9]. We can use TFL to perform vaporization for these small hard nodules to keep the enucleation in the correct surgical plane and decrease the risk of recurrence [7]. Another challenge of AEEP for very large prostates is maintaining proper orientation under endoscopic view. Since it is more difficult to locate the anatomical landmarks in mega-prostates than in those of small or medium-sized. Moreover, hyperplasic glands may protrude into the bladder and block the surgeon’s view of the ureteral orifices [1, 6]. All these factors can increase the risk of complications.

Therefore, we use “Double Rings” En-bloc technique with early anatomical apical release in ThuFLEP for these large glands to avoid the above-mentioned problems. We would like to emphasize here is that the early apical release is anatomical and visual, which is different from the reported “draw white line” technique [10–13]. We combined the cutting of the apical urethral mucosa with the separation of the apical glands into one step. The external urinary sphincter can be well preserved without residual urethral mucosal flaps which may decrease the risk of postoperative SUI. Although our primary data suggest that early apical release approach in ThuFLEP can decrease the risk of postoperative SUI, high-level evidence studies are still lacking. Expanding our sample size with long-term follow-up is our next plan. We prefer to enter the bladder through the 12 o’clock position, as it offers the shortest distance and the safest route, particularly when the median lobe is significantly enlarged. In our approach, the bladder neck serves as a reliable landmark for guiding the dissection of the lateral plane. This technique proves especially advantages in cases of large adenomas with thick anterior tissue, as it allows us to easily create a groove at the 12 o’clock position to reach the bladder neck. This step represents a key distinction from the standard En-bloc technique described by de Figueiredo FCA et al. [14]. So, we called the combination of the apical ring and the bladder neck ring as “Double Rings” technique.

It has been reported in the literature that En-bloc technique may shorten the operative time [15, 16]. En-bloc technique had significantly shorter overall operation time, faster enucleation speed, and shorter enucleation time when comparing with two-lobe/three-lobe techniques [17–19]. However, there were also authors who reported that En-bloc technique had less favorable outcomes in terms of morcellation rate for prostate > 150 cc [18].

We acknowledge that our study has certain limitations, including a small sample size, being a single-center study, and a ~ 25% follow-up loss at the two-year mark. We observed that the mean enucleated weight (70 g) measured postoperatively was relatively low compared to the mean preoperative prostate size (135 mL). Some tissue was vaporized during the enucleation process, which may lead to discrepancies. Similarly, in a large-sample HoLEP study involving 675 patients reported by Assmus, M. A. et al. [20], the average intraoperative tissue weight was 72.1 g, while the preoperative prostate size was 111.2 mL.

Importantly, neither stress urinary incontinence (SUI) nor re-obstruction was observed during the two-year follow-up, further affirming the safety and effectiveness of our technique. Additionally, we believe this approach can be effectively replicated using other energy sources to perform anatomical endoscopic enucleation of the prostate.

## Conclusions

Our presented data show that “Double Rings” En-bloc technique with early anatomical apical release in ThuFLEP seems to be a safe and effective approach for the patients with large glands (≥ 80 mL). However, multi-center and large sample studies are still needed.

## Supplementary Information

Supplementary Material 1.

## Data Availability

No datasets were generated or analysed during the current study.
